# The Effect of a Single Session of Whole-Body Vibration Training in Recreationally Active Men on the Excitability of the Central and Peripheral Nervous System

**DOI:** 10.2478/hukin-2014-0036

**Published:** 2014-07-08

**Authors:** Daria Chmielewska, Magdalena Piecha, Edward Błaszczak, Piotr Król, Agnieszka Smykla, Grzegorz Juras

**Affiliations:** 1 Department of Physiotherapy Basics, Jerzy Kukuczka Academy of Physical Education, Katowice, Poland; 2 Department and Faculty of Medical Biophysics, Medical University of Silesia, Katowice; 3 Department of Human Motor Behavior, Jerzy Kukuczka Academy of Physical Education, Katowice, Poland.

**Keywords:** vibration, chronaxy, excitability, flicker fusion

## Abstract

Vibration training has become a popular method used in professional sports and recreation. In this study, we examined the effect of whole-body vibration training on the central nervous system and muscle excitability in a group of 28 active men. Subjects were assigned randomly to one of two experimental groups with different variables of vibrations. The chronaximetry method was used to evaluate the effect of a single session of whole-body vibration training on the excitability of the rectus femoris and brachioradialis muscles. The examination of the fusing and flickering frequencies of the light stimulus was performed. An increase in the excitability of the quadriceps femoris muscle due to low intensity vibrations (20 Hz frequency, 2 mm amplitude) was noted, and a return to the initial values was observed 30 min after the application of vibration. High intensity vibrations (60 Hz frequency, 4 mm amplitude) caused elongations of the chronaxy time; however, these differences were not statistically significant. Neither a low intensity vibration amplitude of 2 mm (frequency of 20 Hz) nor a high intensity vibration amplitude of 4 mm (frequency of 60 Hz) caused a change in the excitability of the central nervous system, as revealed by the average frequency of the fusing and flickering of the light stimulus. A single session of high intensity whole-body vibration did not significantly decrease the excitability of the peripheral nervous system while the central nervous system did not seem to be affected.

## Introduction

Vibration training, which is also known as NEMES (neuromuscular mechanical stimulation), is a relatively new training practice used in professional sports, physiotherapy and fitness (Cochrane, 2011). According to Gyulai et al. (2013) the reaction to whole-body vibration is training and individual dependent. Many studies have investigated the effects of vibration training on the human body. The biological response of particular types of tissues to applied mechanical vibrations depends on several variables, including the vibration variables, the physiological properties of tissues and individual variation (Jordan et al., 2005). Functional changes following the exposure to whole-body vibration training have been attributed to enhanced neuromuscular function (Junggi et al., 2010).

The reaction of muscles to vibration is a reflex property, defined in the literature as the tonic vibration reflex (TVR) (Eklund and Hagbarth, 1966). According to Cochrane, the tonic vibration reflex requires a vibration with high frequencies (above 100 Hz) to be directly applied to the muscle or tendon (Cochrane, 2011; Cochrane, 2011). When mechanical distension of the muscular-tendon system is initiated, the endings of the Ia annular-spiral myelin fibers are activated, leading to the activation of α motoneurons. As a result, the muscle contracts.

Changes in maximal strength, explosive strength and mechanical power of muscles after vibration training have been described (Jordan et al., 2005). Therefore, to date, the neural mechanisms that are responsible for enhancing muscular performance are still unclear (Cochrane, 2011; Cochrane, 2011).

Densitometric examinations of bones have revealed an increased density of bone tissue after long-lasting vibration training (Ligouri et al., 2012). The results of studies investigating the influence of vibration training on stability of the human body are contradictory and equivocal (Piecha et al., 2013). Vibration training stimulates the process of medication and convalescence; thus, it has become quite popular in rehabilitation. It can also be applied to patients with hemiparesis (Tihanyi et al., 2007). Moreover, there have been several reports in the literature demonstrating that resistance exercises combined with mechanical vibrations prevent atrophy of the muscles when a patient has been immobilized in a lying position for a long period of time (Belavý et al., 2009).

The effects of mechanical vibrations on specific systems and tissues of the human body have been examined by scientists for quite some time (Bosco et al., 2000; Games and Sefton, 2011; Schulte et al., 2011). However, there has been a paucity of reports in the literature regarding the effect of vibration on the excitability of the central nervous system. It has been shown that local application of vibration causes an increase in the excitability of the motor cortex. Furthermore, stimulation of the peripheral sensory entrances by mechanical stimuli can cause an increase in corticospinal excitability in the absence of actual involuntary movements (Forner-Cordero et al., 2008). The increased excitability of the motor cortex supports the process of motor learning, which is an important element of sports training. Changes in the electrical potential of muscles (recorded as an electromyographic signal) caused by a single application of vertical vibration have been examined (Krol et al., 2011; Cardinale and Lim, 2003; McBride et al., 2010). The effect of whole-body vibration at different frequencies (20 Hz, 40 Hz, 60 Hz) and amplitudes (2 mm, 4 mm) upon the medial and lateral vastus muscle in young men has also been evaluated and applied in electromyography. The highest bioelectric activity was observed at a frequency of 60 Hz and a movement amplitude of 4 mm (Krol et al., 2011). Cardinale and Lim (2003) used an electromyographic record of the lateral vastus muscle of a dominant limb and compared the effect of different vibration frequencies in 16 professional volleyball players. The players were exposed to four 60-s vibration applications of different frequencies (30 Hz, 40 Hz, 50 Hz). In addition, the players maintained a static knee-bent position with their knee joints flexed at 100°. It was shown that a vibration of 30 Hz resulted in a 34% increase of the EMG electromagnetic potential, which was significantly different from the results in the control group. A similar observation was also made for the EMG signal, which indicated that there was an increase in the activity of the triceps muscle of a calf up to 8 min after static exercise on a vibration platform (vibration frequency = 30 Hz, deflection amplitude = 3.5 mm). Moreover, analysis of changes in the Hoffman’s reflex did not show any considerable increase in the excitability of the motor neuron (tibial nerve) (McBride et al., 2010).

The main objective of the study was to investigate the effect of whole-body vibration on the excitability of the central nervous system and spinal nerves. The aim of this study was to examine the effect of a single session of whole-body vibration on the excitability of the peripheral and central nervous system in recreationally active men.

## Material and Methods

### Participants

Twenty-eight recreationally active men, who participated in the examinations, were randomly divided into two experimental groups (A and B). The groups differed in the values of the mechanical vibrations induced by the vibration platform. The extreme values of the vertical vibrations from the vibration platform selected for the examinations were 2 mm/20 Hz and 4 mm/60 Hz.

The following criteria were used to qualify the group participants: men between the ages of 19 – 26 who trained twice a week in an amateur football club. Exclusion criteria were: past injuries of the knee joint or the hip joint after operation, anti-pain blockades, acute inflammation of soft tissue and elevated temperature.

Initially there were 31 volunteers, three of whom were excluded from the experiment as they did not meet the inclusion criteria. Twenty-eight participants were randomly allocated to one of two experimental groups. Neither the participants nor the investigators, except the one responsible for vibration training, knew to which group the participants were aligned to. The participants were in a good state of health, without any conditions which could influence the course and the result of the test.

There were 12 men in group A. The participants in group A were subjected to a single whole-body vibration session of 20 Hz frequency and 2 mm vibration amplitude.

There were 16 men in group B. The participants in group B were subjected to a single whole-body vibration session of 60 Hz frequency and 4 mm vibration amplitude.

### Measures

There were four investigators responsible for performing different interventions (enrollment of the participants, whole-body vibration training, chronaximetry, Critical Flicker Frequency measurement). The measurements were taken in late morning hours to minimize the impact of daily tiredness. The subjects were asked not to eat or drink food containing caffeine two hours before measurements and not to take up intensive physical exercises 24 hours before the measurements. The measurements were made under standard conditions, which were the same for all subjects. These examination methods were approved by the Bioethics Committee at the Academy of Physical Education in Katowice (12/2008).

Changes in the critical values of the fusing and flickering frequencies of optical stimuli (Critical Flicker Frequency – CFF) due to two vibration intensities before and after a vibration session were tested to examine the effect of mechanical vibrations on the excitability of the central nervous system. The effects of a single whole-body vibration session of two intensity levels on the chronaxy values (Chr) of the rectus femoris (engaged directly in the exercise) and brachioradialis muscle (engaged indirectly in the exercise because of its peripheral position) were examined. Chronaxy measurements were made before the exercise session, up to 2 min after the application and 30 min following the application.

The FitVibe 600 vibration platform (GymnaUniphy N.V) had been used in previous studies (Piecha et al., 2013). The characteristics of the platform vibrations were a shift in the vertical direction, a vibration frequency ranging from 20 to 60 Hz, a low (2 mm) or high (4 mm) vibration amplitude and a time of vibration that ranged from 1 to 90 s.

### Procedures

The subject remained in a static position during the exercises. A test team member stood on the surface of the platform and loaded it uniformly. The hip and knee joints were flexed at 90°, and the upper limbs were placed against the pelvis. This position mainly engaged the gluteus maximus muscles and the quadriceps femoris muscles. Importantly, the procedure was safe because the flexion of the knee joints reduced the transfer of vibrations via spine and into the head. In case of platforms that operated in the vertical mode, the increase in bioelectrical activity of the lower limbs in the position where the knee joints flexed at 90° was noted (Krol et al., 2011). The exercise and rest periods lasted 1 min, in accordance with the procedure used in previous studies (Bosco et al., 2000). The exercises were repeated 5 times.

The examination of the fusing and flickering frequencies of the light stimulus (Critical Flicker Frequency) made it possible to analyze the response to a light wavelength of 650 nm when perceived by the tested person as a fixed light. The increasing and decreasing frequency was in the range of 25 Hz – 60 Hz, and a rate of change of 1 Hz/s was used in the examinations. The examination started by generating a stimulus frequency of 60 Hz, which was perceived as a fixed light. The frequency was then reduced until the tested person signaled (by pressing a button) that he noticed a flickering light (flicker threshold). In the second sequence of measurements, the initial frequency was 25 Hz, which was then increased (to a maximum of 60 Hz) until the tested person observed a fixed light (Fusion Threshold). Eight measurement cycles were performed, and the mean values of all of the registered frequency measurements were calculated.

The chronaxy measurements were performed using an Intelect advanced device (Chattanooga). Chronaximetry is a quantitative diagnostic method for neuromuscular conductivity that is used in physiotherapy. Chronaxy is defined as the shortest impulse time for a rectangular course at the intensity value of a double rheobase, which generates a minimal contraction of a skeletal muscle. The skin where the electrodes were attached was cleaned with alcohol. A point electrode connected to the negative end of an interrupted direct current was applied to stimulate the direct motor points of the rectus femoris and brachioradialis muscles to determine the chronaxy. Chronaxy measurements were obtained before, immediately after, and 30 minutes following the vibration session. This procedure was in accordance with the recommendations of Leslie Geddes (2004), and the chronaxy values obtained were in the range of 0.01 ms and 1 ms, indicating normal neuromuscular excitability (Nelson, 1998).

### Statistical analysis

The obtained data were validated by commonly used methods of statistical analysis. The Shapiro-Wilk test was used to verify the data’s compatibility with a normal distribution. The homogeneity of variance was evaluated by the Levene test. However, not all of the variables met the assumption of normality of the variable distribution and homogeneity of variance; therefore, the Wilcoxon matched pairs test (confidence level ≤ 0.05) was used to compare the mean values of the chronaxy before and after the session of vibration exercise and 30 minutes later to compare the relative changes of the chronaxy between measurements 1 and 2, 1 and 3, and 2 and 3 in groups A and B. This test was also used to compare the mean frequency values of the fusing and flickering frequencies of the light stimulus. The Mann-Whitney U test was used to compare these parameters between groups A and B.

## Results

Group A included 12 men with a mean age of 21.6 ± 1.35 years, body mass of 75.2 ± 5.81 kg and a BMI of 22.54 ± 1.34. Group B was composed of 16 men with a mean age of 21.63 ± 1.2 years, body mass of 74.63 ± 5.77 kg and a BMI of 23.76 ± 1.75. The test groups were homogeneous for anthropometric features, and the tested men were characterized. There were no obvious changes in the mean frequency of the fusing and flickering of the light stimuli due to the application of different vibration intensity (Table 1).

The obtained chronaxy values for all of the members of the test groups before and after the application of vibration were within standard norms; i.e., they were lower than 1 ms. The comparison of the mean chronaxy values of a rectus femoris (this muscle was directly engaged) before vibration (measurement 1) and after vibration (measurement 2) and between the value after vibration and 30 min later (measurement 3) in group A showed that they were significantly different. The mean chronaxy value decreased due to low intensity training. Thirty minutes after the application of vibration, the mean value of the chronaxy approximated its value before training (Table 2). The change of the excitability of the quadriceps femoris muscle under the influence of a single whole-body application of vibration was reversible (Table 2). In addition, the relative improvements of the chronaxy after training compared to its value before the application and 30 min later were 15.44% and 4.94%, respectively.

The mean chronaxy values of the quadriceps femoris muscle of the thigh under the influence of high-intensity vibration in group B increased immediately after the exercise session, but these changes were not statistically significant (Table 2).

The chronaxy results of the brachioradialis muscle, which was positioned peripherally against the lower limbs and was directly involved in the exercise, did not show statistically significant changes (Table 2).

No differences were found between groups A and B with regard to any of the examined variables (Tables 1 and 2).

Neither adverse events nor side effects were observed after interventions in each experimental group.

## Discussion

Several novel training tools have been identified to optimize the effects of motor exercises. Therefore, there have been no examinations of the effects of mechanical vibrations on the excitability of the neuromuscular system, prompting an investigation of this subject.

Results from the use of chronaximetry to evaluate the excitability of the neuromuscular system have been previously described in the literature (Kiernan et al., 2000; Murray and Jankelowitz, 2011; Ng and Burke, 2007).

Chronaximetry is the first method of 4 presented in the examination protocol for the conductivity of peripheral nerves, as described by Bostock et al. (1998). The simplicity and reliability of the protocol (including chronaximetry) have been confirmed in the course of examinations for evaluation of the excitability of the median and ulnar nerves in healthy subjects (Kiernan et al., 2000; Murray and Jankelowitz, 2011). The higher values of the amplitude and time of electrical stimulus that are essential to generate excitability from a median nerve (compared to the results obtained for the ulnar nerve) were observed. In addition, the authors highlighted the differing threshold excitability of the examined nerves in healthy subjects (Murray and Jankelowitz, 2011).

Mogyoros et al. (1996) repeatedly noted the dependence of neuromuscular excitability on the time of electrical impulse in successive examinations performed on 20 healthy subjects. In other types of examinations, convergence of a quantitative evaluation of the skeletal muscle denervation was obtained by applying chronaximetry and needle electromyography methods (EMG). In case of an acute phase of denervation and a total degenerative reaction, the sensitivity of both methods was 100%. The convergence of the EMG diagnostic results and chronaximetry was 86% at some time after the injury and partial degradation reaction (Paternostro-Sluga et al., 2002).

Currently, there have been no publications on the evaluation of the effects of vibration on muscle or nerve excitability using chronaximetry. Two vibration frequencies were used, 20 Hz and 60 Hz, and the vibration amplitudes were 2 mm and 4 mm, which resulted in diversified effects. The use of lower parameters in a single vibration session (20 Hz/2 mm – group A) caused a significant decrease in the chronaxy value of a quadriceps femoris muscle after the application of whole-body vibration. When high values of the vibration parameters (60 Hz/4 mm – group B) were used, elongation of the chronaxy time of this muscle was observed; however, these changes were not statistically significant.

Although there is strong evidence that the chronaximetry method is reliable for assessing the neuromuscular system, we question whether it is sensitive enough for use in healthy subjects.

There were no statistically significant changes in the brachioradialis muscle excitability since it was not exposed directly to vibrations because of the position of the exercise and the peripheral position of the muscle. In whole-body vibration training, mechanical vibrations are transmitted from the distal parts, which are in contact with the platform in the proximal direction (Luo et al., 2005). In the course of the transmission of mechanical vibrations in human tissues, the frequency, amplitude and intensity of the vibration are reduced due to the damping properties of soft tissues (Cardinale and Wakeling, 2005).

Hazel et al. (2007) did not observe a change in the activity of the biceps muscle in the course of static exercises performed on a platform at a maximum frequency of 45 Hz. Thus, the following initial position of the exercise was recommended: direct hand contact with a vibration platform or the application of a point vibration to activate the muscles of the upper limbs. Bosco et al. (2000) showed that the bioelectrical activity of the biceps muscle doubled during the application of a point vibration of 30 Hz frequency and 6 mm amplitude to the tense muscle.

Previous study results demonstrated varying effects of vibration training on the human body, which can depend on the parameters of the mechanical vibrations. The vibration amplitude of the platforms ranged from 1 to 10 mm. In the literature, the vibration frequencies used for exercises were within the range of 20 to 60 Hz. Mechanical vibrations of lower frequencies than 20 Hz were not used because they initiated the phenomenon of mechanical resonance, which can be harmful for organs (Jordan et al., 2005). When the vibration frequencies are higher than those commonly used in vibration training, the excess of stimuli for neuromuscular spindles and sensory receptors can disturb the evaluation of afferent information by the central nervous system.

It has been suggested that the constant application of vibration lasting longer than one minute can cause muscle fatigue (Jordan et al., 2005). In these examinations, we chose to maintain the same position on the platform for one minute. Krol et al. (2011) similarly performed their examinations on healthy subjects. Tihanyi et al. (2007) applied a single whole-body vibration to patients with past stroke conditions for one minute (on a platform, repeated 6 times) and used a frequency of 20 Hz with an amplitude of 5 mm. The achieved isometric and eccentric increase of the moment of force of the knee extensor muscles and the growth of the EMG amplitude of the vastus lateralis muscle by 44.9% was statistically significant. There were no considerable changes in the control group (placebo vibration).

The effects of whole-body vibration on the excitability of the central nervous system have not been previously presented.

Transcranial magnetic simulation revealed several changes in the motor cerebral cortex due to the local vibration of the upper limb. Forner-Cordero et al. (2008) showed that if muscle tendons of the wrist extensors and flexors were simulated by vibrations of 80 Hz frequency for a long time, corticospinal excitability increased. According to the authors, one possible explanation for the obtained results may be that the cerebral cortex features high reorganizational abilities. The motor and sensory cortices of a healthy adult have the ability to react rapidly to any changes in the external environment. Such neuroplasticity is connected with permanent, functional and structural changes in the cerebral cortex as a response to active stimuli (Donoghue, 1995).

The light fusing and flickering test (CFF) was used to evaluate the activity of the central nervous system and sensory sensitivity of the examined men. The frequencies at which flickering (still > flickering) and fusing (flickering > still) of the point light source occur are the criteria that indicate excitability of the central nervous system.

Physical exercise has an effect on the increase of the critical value of the fusing frequency of the light stimuli. Changes in the CFF test index show increases in the excitability of the cerebral cortex (cortical excitability). Thus, this test may be used to assess the degree of central nervous system fatigue (Davranche and Pichon, 2005). In other types of examinations, Davranche et al. (2005) subjected physically active young men to exercises on a cycloergometer with a load of 50% VO_2max_. There was an increase in the critical flicker frequency (CFF) directly after the exercises, which, according to the authors, indicated an increase of sensory sensitivity.

In the present study, we did not find any significant changes in the fusing and flickering frequencies of the light stimuli in a single whole-body vibration session of varied intensity. However, in some cases, there was a slight (but statistically insignificant) increase of the mean value of the light fusing frequency as well as a slight decrease of the light flickering frequency. However, this was only found in group A. On the basis of the presented data, we presume that the activity of the central nervous system increased when the vibrations were applied, but such conclusions were not confirmed by the obtained results.

Men between the ages of 20 – 24 participated in the present study. The nervous system at that stage of ontogenetic development is characterized by a relative balance between stimulation and inhibition processes. In addition, the level of coordination abilities of all of the examined subjects was very high. We propose that the lack of significant differences in the chronaxy values after vibration training in the examined group is related to the high excitability observed in healthy subjects. According to Dolny and Reyes (2008), the exposure to whole-body vibration in young and healthy people is an adequate stimulus for the generation of adaptive changes in the neuromuscular system.

Games and Sefton (2011) stated that there had not been any studies on the effect of a whole-body vibration on the central and peripheral nervous system, which (according to the authors), is quite fundamental as far as the application of this method in elderly and neurological patients.

We present evidence to suggest that the action of acute vertical vibration on muscle enhances strength, power, balance and proprioception. Little attention has been given to the neural mechanisms of whole-body vibration training. However, our current conclusions, which are based on the performed examinations, suggest that a single session of whole-body vibration training may have an effect on the excitability of the peripheral nervous system. During this study, changes in the central nervous system excitability were not observed. The effects of whole-body vibration training on the nervous system with regard to its implications on neurological rehabilitation and for elderly patients are interesting. We are aware of a demand for further investigation of the effects of different forms of vibration training on the nervous system, as well as on central and peripheral excitability.

## Key Points

1. The application of whole-body vibration with diversified parameters did not change the activity of the central nervous system in any of the two groups examined.
2. A single whole-body vibration session of 20 Hz frequency and 2 mm amplitude generated a considerable decrease in the chronaxy value of the quadriceps femoris muscle, which was directly engaged in the training, to a value similar to its value prior to the vibration application.
3. A whole-body vibration frequency of 60 Hz and an amplitude of 4 mm did not generate statistically significant changes in the chronaxy of the quadriceps femoris muscle.
4. No changes in the chronaxy value of the brachioradialis muscle were noted when the vibration was applied.

Our findings seem to indicate that a single whole-body vibration session does not change the excitability of the neuromuscular system in healthy men. Therefore, whole body vibration can safely be used as a part of athletic training. However, further studies of larger populations are needed to explore the influence of various types of whole-body vibration training on the neuromuscular system.

## Figures and Tables

**Table 1:** Comparison of the mean values of fusing and flickering frequency of the light stimulus before and after a single whole-body vibration session in group A and B.

**Measured variable**	**Group**	**Number of participants**	**1 - mean value before vibration [Hz] ± SD**	**2 - mean value after vibration [Hz] ± SD**	p^*^1, 2	p^**^A, B
**Frequency of fusing**	A	12	38,54 ± 2,79	39,54 ± 2,64	p>0,05	p>0,05
	B	16	36,77 ± 3,02	36,97 ± 4,07	p>0,05	
**Frequency of flickering**	A	12	44,61± 3,17	43,62 ± 2,95	p>0,05	p>0,05
	B	16	43,64 ± 2,38	43,70 ± 4,15	p>0,05	

* Wilcoxon test

** Mann-Whitney U test

**Table 2:** Comparison of the mean values of chronaxy before, after and 30 min later after a single session of a whole-body vibration in group A and B

**Tested muscle**	**Group**	**Number of parcicipants**	**1 - mean value of Chr. before [ms] ± SD**	**2 - mean value of Chr. after [ms] ± SD**	**3 - mean value of Chr. after 30 min. [ms] ± SD**	**p** 1, 2^*^	**p** 2, 3^*^	**p** A, B^**^
**Rectus femoris muscle**	A	12	0,140 ± 0,07	0,116 ± 0,05	0,141 ± 0,05	p=0,05	p=0,02	p>0,05
	B	16	0,170 ± 0,12	0,211 ± 0,21	0,188 ± 0,09	p>0,05	p>0,05	
**Brachio- radialis muscle**	A	12	0,163 ± 0,09	0,168 ± 0,13	0,175 ± 0,15	p>0,05	p>0,05	p>0,05
	B	16	0,273 ± 0,24	0,272 ± 0,24	0,228 ± 0,22	p>0,05	p>0,05	

* Wilcoxon test

** Mann-Whitney U test
